# Efficacy of the U‐shaped flap technique in preventing reflux after minimally invasive proximal gastrectomy for proximal gastric and esophagogastric junction cancer

**DOI:** 10.1002/ags3.12864

**Published:** 2024-10-09

**Authors:** Takeshi Omori, Hisashi Hara, Yoshitomo Yanagimoto, Naoki Shinno, Yasunori Masuike, Takashi Kanemura, Hiroshi Wada, Masayoshi Yasui, Masayuki Ohue, Hiroshi Miyata

**Affiliations:** ^1^ Department of Gastroenterological Surgery Osaka International Cancer Institute Osaka Japan; ^2^ Department of Gastroenterological Surgery Osaka International Medical and Science Center Osaka Japan

**Keywords:** double flap, esophagogastric junction carcinoma, esophagogastrostomy, gastric cancer, reflux esophagitis, single flap, U‐shaped flap

## Abstract

**Background:**

Preventing gastroesophageal reflux after proximal gastrectomy for proximal gastric and esophagogastric junction cancer remains challenging due to the lack of standardized reconstructive techniques. The double flap technique (DFT) in valvuloplastic esophagogastrostomy prevents reflux esophagitis but is less effective in esophagogastric junction cancer because of negative pressure on the inferior mediastinum. We developed the U‐shaped flap technique (UFT) to enhance the anti‐reflux efficacy.

**Methods:**

This study analyzed data from patients who underwent minimally invasive proximal gastrectomy for proximal gastric and esophagogastric junction cancer between August 2014 and May 2022, using a prospectively maintained database. We compared DFT and UFT for short‐ and long‐term outcomes, focusing on gastroesophageal reflux, using one‐to‐one propensity score matching to control for patient‐related variables.

**Results:**

Among 217 eligible patients, 205 (100 in DFT, 105 in UFT) completed a 1‐year follow‐up. After propensity score matching, we selected 42 pairs of patients who underwent DFT and UFT. UFT had significantly shorter operative time (*p* = 0.044), similar blood loss, and similar morbidity. The UFT group had significantly fewer reflux symptoms (0% vs. 14.3%, *p* = 0.0011) and endoscopic Los Angeles grade B or higher reflux esophagitis (0% vs. 14.3%, *p* = 0.0011) than the DFT group. In lower mediastinal reconstructions for esophagogastric junction cancer, UFT showed a reduced incidence of reflux esophagitis.

**Conclusion:**

Our study indicates that the U‐shaped flap technique (UFT) offered significant advantages in reducing postoperative reflux symptoms and endoscopic esophagitis, in a cohort of patients with proximal gastric and esophagogastric junction cancer.

## Graphical abstract

The U‐shaped flap technique (UFT) is highly effective in reducing reflux symptoms and esophagitis after proximal gastrectomy for upper gastric cancer, especially esophagogastric junction cancer.

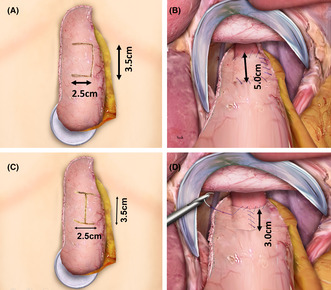

## 1 INTRODUCTION

Gastric cancer is the fifth most prevalent cancer worldwide and the fourth leading cause of cancer‐related mortality, posing a significant global health challenge.^1^ Recent years have seen a notable increase in the incidence of proximal gastric and esophagogastric junction cancers.^2^, ^3^ Reflecting the trend toward minimally invasive and function‐preserving surgical approaches, laparoscopic proximal gastrectomy has become the preferred treatment for early‐stage proximal gastric cancer. This method is favored for its numerous advantages over total gastrectomy, including reduced postoperative weight loss, enhanced quality of life, and better preservation of hemoglobin and vitamin B12 levels.^4^, ^5^

The ideal method for reconstruction after proximal gastrectomy remains a topic of debate. Esophagogastrostomy is commonly chosen for its advantages, such as maintaining the duodenal passage, ease of anastomosis, direct observation of the residual stomach, and minimal nutritional impact.^2^, ^3^, ^6^ However, this reconstruction has the risk of inducing reflux esophagitis due to gastric acid reflux, which greatly affects the patient's quality of life.^1^, ^6^, ^7^

Addressing this issue, the double flap reconstruction technique (DFT), a modified form of valvuloplastic esophagogastrostomy introduced by Kamikawa, aims to prevent gastroesophageal reflux post‐gastrectomy.^8^ This technique involves encasing the distal esophagus and anastomosis site between the submucosal and serosal layers of the residual stomach with an H‐shaped flap, creating a one‐way valve mechanism against reflux. However, the DFT involves a central incision in the gastric serosal flap, which can complicate the procedure and increase the risk of complications such as suture dehiscence and structural weakness. While effective in early‐stage proximal gastric cancer cases,^7^, ^8^, ^9^, ^10^ its efficacy in esophagogastric junction cancer, particularly in lower mediastinal reconstructions following lower esophagectomy and proximal gastrectomy, has been questioned due to a higher incidence of reflux esophagitis.^9^

To address these challenges, we developed the U‐shaped flap technique (UFT) as an alternative approach. The UFT eliminates the central incision, creating a single continuous flap that wraps around the esophagus. This design not only simplifies the flap creation process but also aims to provide a stronger and more consistent anti‐reflux barrier. This study examines the safety and efficacy of using the U‐shaped flap technique in esophagogastrostomy for treating proximal gastric and esophagogastric junction cancer, following laparoscopic or robotic proximal gastrectomy/lower esophagectomy.

## 2 METHODS

### 2.1 Study design and data collection

This study was conducted as a single‐institute retrospective study utilizing a database that was prospectively collected. All data were gathered from patients who underwent proximal gastrectomy at our institution. The database includes detailed information on patient demographics, surgical procedures, postoperative outcomes, and follow‐up data.

We hypothesized that the U‐shaped flap technique is superior to the double flap technique with regard to regurgitation and endoscopic reflux esophagitis at 1‐year follow‐up. This study design was reviewed and approved by the institutional review board of Osaka International Cancer Institute (Protocol ID: 18070‐3). All procedures performed in studies involving human participants were in accordance with the ethical standards of the institutional and/or national research committee and with the 1964 Helsinki Declaration and its later amendments or comparable ethical standards.

### 2.2 Informed consent

Informed consent, in written form, was secured from all patients. Before undergoing surgery, these patients were thoroughly informed about the nature of the study, its purpose, the associated surgical and oncological risks, and the use of their data for research purposes. This process ensured that all ethical guidelines and regulations were strictly followed.

### 2.3 Patients

**FIGURE 1:**
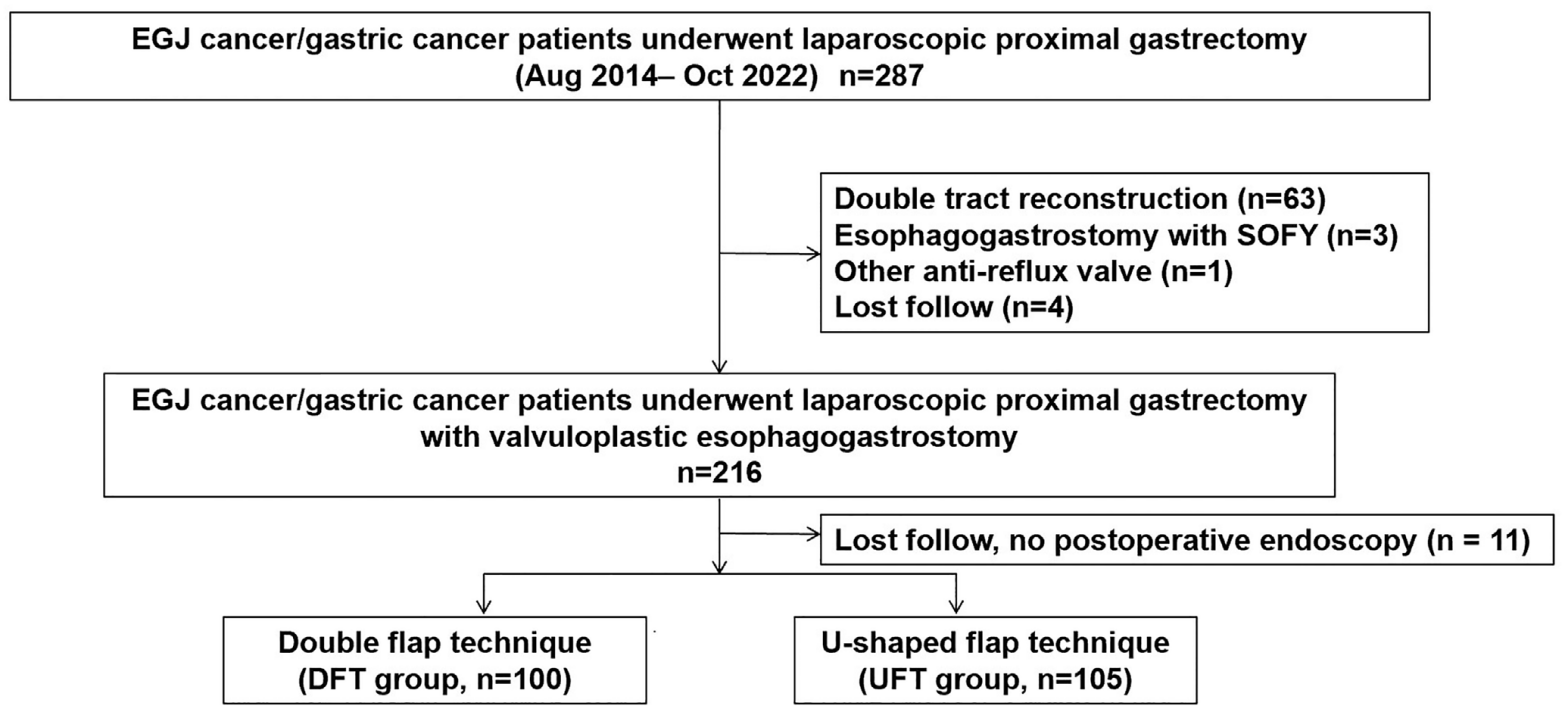
Patient flow chart.

From August 2014 to October 2022, laparoscopic proximal gastrectomy (LPG) was performed on 287 patients with proximal gastric cancer (PGC) or esophagogastric junction carcinoma (EGJC), classified according to the Siewert classification,^11^ at the Osaka International Cancer Institute in Osaka, Japan. This cohort was identified through a review of our prospective gastric cancer database. Because this study was conducted to examine better valvuloplastic esophagogastrostomy procedures, patients who underwent other anti‐reflux procedures such as SOFY were excluded from this study. The indication for esophagogastrostomy was that the residual stomach should remain more than half of the stomach. If less than half of the stomach remained, double tract reconstruction was performed. Esophagogastrostomy with the double flap technique for treating proximal gastric cancer was introduced at our hospital in August 2014. In February 2019, we developed the U‐shaped flap technique to facilitate the valvuloplastic procedure, which has since become the preferred method of treatment at our institution. The eligibility criteria for this study included: (1) being over 20 years of age, (2) having histologically confirmed malignant tumors, specifically proximal gastric cancer, esophagogastric junction (EGJ) cancer, gastrointestinal stromal tumors (GIST), or liposarcoma, and (3) having undergone laparoscopic or robotic proximal gastrectomy, or lower esophagectomy and proximal gastrectomy. Patients with a history of abdominal surgery and those with R1 resection due to positive peritoneal lavage cytology, yet without evident gross peritoneal dissemination, were also eligible. This inclusion criterion was based on our routine practice of performing gastrectomy for stage IV gastric cancer classified as P0CY1. Conversely, patients presenting with bulky lymph nodes or distant metastases, such as paraaortic lymph nodes, liver metastases, or peritoneal dissemination, received chemotherapy before surgical intervention. Furthermore, the study included patients who underwent neoadjuvant chemotherapy and those who received conversion surgery after chemotherapy for previously unresectable gastric cancer. Ultimately, 216 patients with PCG or EGJC who received laparoscopic proximal gastrectomy accompanied by valvuloplastic esophagogastrostomy were identified. Of these, 205 patients who completed a 1‐year follow‐up were included in this study (Figure 1).

### 2.4 Gastrectomy with Lymphadenectomy

In this study, all procedures were performed by a single surgeon who is certified in laparoscopic gastrectomy and has performed over 100 laparoscopic/robotic surgeries. Preoperatively, indocyanine green (ICG) endoscopic marking was applied as previously described.^12^ Patients were positioned in reverse Trendelenburg, with leg position varying between laparoscopic (open legs) and robotic (closed legs) approaches. A 2.5–3.0 cm vertical umbilical incision was made for transumbilical laparotomy, followed by the application of a wound‐sealing device and a commercial access port (Lap Protector and EZ access; Hakko, Nagano, Japan). Carbon dioxide gas insufflation at 8–12 mmHg created pneumoperitoneum, with surgery performed using a conventional five‐ or six‐port setup. In laparoscopic proximal gastrectomy (PG), ultrasonic coagulating shears, either Harmonic 1100 (Ethicon Endosurgery, Cincinnati, OH) or Sonicision™ (Medtronic, Dublin, Ireland), were utilized for gastric mobilization and lymph node dissection. In robotic procedures, Maryland bipolar forceps equipped with soft coagulation mode were primarily employed.^13^ Lymph node dissection extended according to the 15th edition of the Japanese Classification of Gastric Carcinoma^14^, ^15^: D1+ lymph node dissection (nodes 1, 2, 3a, 4sa, 4sb, 7, 8a, 9, and 11p) for proximal gastric cancer, and additional nodes for early and advanced esophagogastric junction cancer.

Surgical steps included: (1) dissection of the greater omentum and No. 4sa/4sb lymph nodes, preserving the right gastroepiploic artery; (2) perigastric lymph node dissection (nos. 1, 2, 3a), preserving right gastric vessels; (3) suprapancreatic node dissection (nos. 7, 8a, 9, 11p, 11d); (4) transection of the distal stomach using a linear stapler, ensuring a margin over 20 mm from the lesion; and (5) esophageal resection with a similar margin. For esophagogastric junction cancer, post‐abdominal lymphadenectomy included cutting the diaphragmatic crus for lower mediastinal access and transhiatal dissection of lymph nodes (nos. 110, 111, 112). Resected specimens were extracted via the umbilical incision.

In both the U‐shaped flap technique (UFT) and the double flap technique (DFT), the upper edge of the gastric remnant flap is sutured to the posterior wall of the esophagus 5 cm oral from the anastomosis. In the UFT, the flap is wrapped around the esophagus in a U‐shape, effectively covering a 5 cm span. In contrast, in the DFT, the flap is wrapped in a V‐shape, covering a 3 cm span.

#### 2.4.1 U‐shaped flap reconstruction (a novel technique) (Figure 2)

**FIGURE 2:**
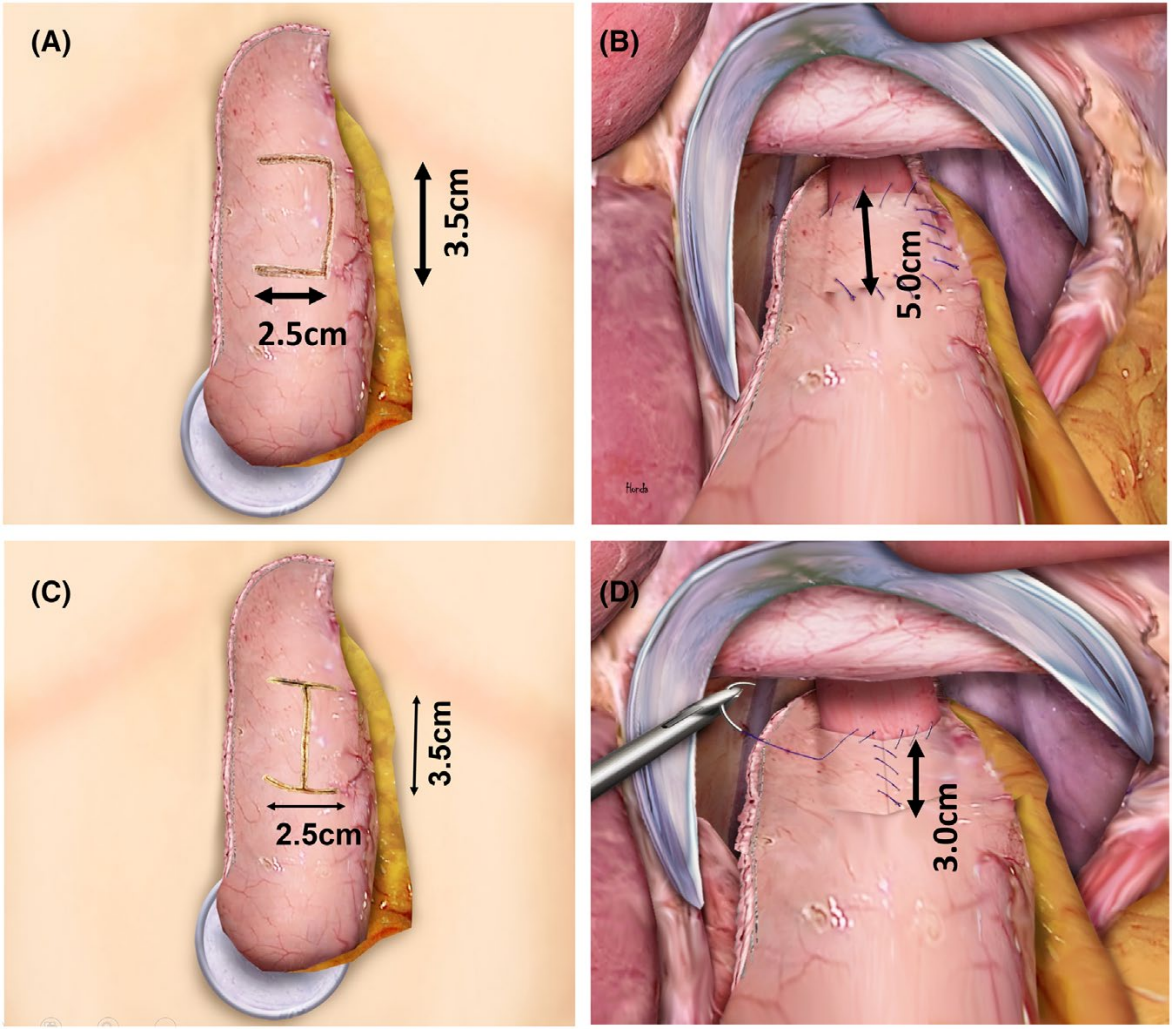
Scheme of U‐shaped flap and double flap technique. (A) U‐shaped flap was created on the anterior wall of the gastric remnant (2.5 cm × 3.5 cm). (B) The U‐shaped flap was closed with barbed sutures to cover the distal end of the esophagus, extending 5 cm proximally, similar to a crew‐neck shirt. (C) Double flap was created on the anterior wall of the gastric remnant (2.5 cm × 3.5 cm). (D) The double flap was secured with barbed sutures to cover the distal end of the esophagus, extending 3 cm proximally, similar to a low‐cut V‐neck shirt.

A U‐shaped seromuscular flap, measuring 2.5 cm in width and 3.5 cm in height, was fashioned on the anterior wall of the stomach (Figure 2A). Esophagogastrostomy was then performed at the distal end of the residual stomach's mucosal window. This procedure can be conducted either by hand suturing or using a stapler, which may be linear or circular.^16^, ^17^, ^18^, ^19^, ^20^, ^21^ Afterward, the flap was meticulously closed and wrapped around the anastomotic site and the proximal 5 cm of the distal esophagus, simulating a round‐necked shirt collar. This was achieved using an absorbable barbed suture such as V‐Loc (Medtronic, Dublin, Ireland) or STRATAFIX Spiral (Ethicon Endosurgery, Cincinnati, OH) (Figure 2B). Notably, this technique allows for a longer embedding distance for the esophagus compared to traditional methods.

#### 2.4.2 Double flap reconstruction (traditional) (Figure 2)

H‐shaped seromuscular flaps, each measuring 2.5 cm in width and 3.5 cm in height, were created on the anterior wall of the remnant stomach (Figure 2C). The esophagogastrostomy was performed intracorporeally either manually using barbed sutures or with a stapled anastomotic technique.^17^, ^19^, ^21^ The anastomotic site, along with a 3 cm section proximal to the distal esophagus, was covered by the flap in a V‐neck short fashion using barbed sutures (Figure 2D).

### 2.5 Anastomotic techniques

Two different anastomosis methods were employed in this study: end‐to‐end anastomosis and side‐to‐side anastomosis. The end‐to‐side anastomosis was performed using either hand‐sewn suturing or a circular stapler. The side‐to‐side anastomosis was performed using a linear stapler.

### 2.6 Esophagogastrostomy by hand suture

The procedures were described in the previous report.^17^ Briefly, the posterior wall of the esophagus and the gastric mucosa were sutured together using continuous barbed sutures, specifically using absorbable barbed sutures such as V‐Loc (Medtronic, Dublin, Ireland) or STRATAFIX Spiral (Ethicon Endosurgery, Cincinnati, OH). The anterior wall of the esophagus and the gastric mucosa along the inferior margin of the flap were then sutured in a single, full‐thickness layer using continuous barbed sutures to complete the esophagogastrostomy.

### 2.7 Hybrid methods for valvuloplastic reconstruction

#### 2.7.1 Circular stapled esophagogastrostomy

The procedures were described in the previous report.^20^ Briefly, a flap was created on the gastric wall, followed by an incision 3 cm below the gastric mucosa window's distal end. A circular stapler was used for esophagogastrostomy at the window's distal end, and the anastomotic site and adjacent esophagus were covered with the flap using barbed suture by hand suturing.^19^, ^20^

#### 2.7.2 Linear stapled esophagogastrostomy

The procedures were described in the previous report.^21^ Briefly, esophagogastrostomy was performed using a linear stapler through entry holes on the esophageal stump and below the gastric flap. The anastomotic entry hole was closed with a stapler^21^ or hand stitches.

In all methods, a drain was placed along the suprapancreatic margin and an additional bilateral thoracic drain was placed in patients with esophagogastric junction cancer.

### 2.8 Clinical variables and postoperative management

Data were prospectively collected and recorded in a computer database at the Osaka International Cancer Institute. Perioperative management protocols were consistent for both groups, adhering to our clinical pathway. All patients in this study received proton pump inhibitor (PPI) therapy postoperatively. Postoperatively, patients were followed up routinely every 3–6 months in accordance with the Japanese Gastric Cancer Treatment Guidelines, 6th edition.^15^ Data collected and analyzed included sex, age, histological type, clinical TNM Staging, and pathological TNM stage, as per the 8th edition of the Union for International Cancer Control (UICC) TNM Classification of Malignant Tumors. Surgical data such as surgical approach and R status were also evaluated. Surgical outcomes and complications were systematically assessed. Complications, including anastomotic leakage, pancreatic fistula, and abdominal abscess, were diagnosed through radiographic and clinical evaluations and graded according to the Clavien–Dindo classification.^22^ Body weight loss and nutritional parameters, namely hemoglobin, lymphocyte count, total protein, albumin, and the prognostic nutritional index (PNI), were calculated using the formula: [10 × serum albumin (g/dL) + 0.005 × total lymphocyte count].^23^ Gastroesophageal reflux was assessed 1 year postoperatively using upper gastrointestinal endoscopy and the Visick score.^24^

### 2.9 Propensity score matching analysis

Propensity score matching (PSM) was utilized to minimize confounding variables and mitigate potential selection bias in the retrospective design of this study. Regression models were developed using pertinent variables associated with treatment selection, including age, sex, body mass index (BMI), American Society of Anesthesiologists (ASA) physical status, preoperative levels of hemoglobin and serum albumin, clinical stage, type of esophagogastrostomy (end‐to‐side and side‐to‐side anastomosis), surgical approach (robotic or laparoscopic), and use of preoperative chemotherapy. A 1:1 nearest neighbor matching algorithm, with an optimal caliper width of 0.2, was employed to align the propensity scores. To assess the balance of covariates before and after propensity score matching (PSM), we calculated the standardized differences for each covariate. An absolute standardized difference of 0.1 or below was considered indicative of sufficient balance between the groups.

### 2.10 Statistical analysis

We performed all statistical analyses using version 16 of JMP software (SAS Institute Inc., Cary, NC, USA). Demographic and clinicopathological characteristics were summarized descriptively. Quantitative data were reported as either means ± standard error or medians with interquartile ranges. Student's *t*‐tests and Mann–Whitney *U* tests were used to compare continuous variables, while Pearson's *χ* ^2^ tests were applied for categorical comparisons. All tests were two‐tailed, with a *p*‐value threshold of <0.05 indicating statistical significance.

To identify risk factors for Los Angeles grade B or higher reflux esophagitis, univariate and multivariate logistic regression analyses were performed. The analysis included factors such as age, sex, BMI, surgical approach (robot‐assisted vs. laparoscopic), type of esophagogastrostomy (handsewn vs. stapled), type of anastomosis (end‐to‐side vs. side‐to‐side), type of valvuloplastic esophagogastrostomy (VEG: UFT vs. DFT), and the presence of esophagogastric junction (EGJ) cancer or proximal gastric cancer (PGC). Factors with a *p*‐value less than 0.05 in the univariate analysis were included in the multivariate analysis to control for potential confounding. Odds ratios (OR) with 95% confidence intervals (CI) were calculated, and a *p*‐value of <0.05 was considered statistically significant.

A Forest plot was constructed to assess the odds of postoperative GERD (LA grade B or higher) for UFT versus DFT across various patient subgroups. The plot represents the odds ratios (ORs) with 95% confidence intervals (CIs) to evaluate the relative odds of GERD incidence. Subgroup analysis was stratified by patient demographics (sex, age), surgical approach (laparoscopic vs. robotic), tumor stage (clinical T‐stage, nodal status), overall clinical stage, tumor type (EGJC), and type of anastomosis (end‐to‐side vs. side‐to‐side). Odds ratios were derived using a logistic regression model to estimate the odds of developing significant GERD symptoms. An OR less than 1 indicates lower odds of GERD associated with UFT as compared to DFT. The 95% CIs are depicted as horizontal lines extending from the central point estimate, which signifies the OR for the subgroup. The size of the point reflects the number of patients in the subgroup, with larger points indicating a higher sample size contributing more weight to the meta‐analysis. The plot displays ORs as points where the size indicates the subgroup's sample size and the horizontal line represents the 95% CI. The vertical line at OR = 1 serves as the null effect reference, showing no difference in the odds of GERD between the two techniques. The position of the CIs in relation to this line informs the degree of difference in odds.

## 3 RESULTS

### 3.1 Patient characteristics

Table 1 shows the demographics of the full (*n* = 205) and propensity score matched (*n* = 84) cohorts. The initial cohort included 100 patients in the DFT group and 105 in the UFT group. The gender difference between the DFT and UFT groups was significantly imbalanced (male/female: 86/14 vs. 73/32, *p* = 0.0047). There was also a marked difference in surgical approach, with laparoscopic surgery predominating in the DFT group versus robotic surgery in the UFT group (85/15 vs. 32/73, *p* < 0.0001). The method of esophagogastrostomy also showed a significant difference (hand‐sewn/stapled anastomosis: 22/78 in the DFT group vs. 9/96 in the UFT group, *p* = 0.0073). Clinical T status (cT1/T2/T3/T4a: 57/18/21/4 for DFT vs. 60/8/21/16 for UFT, *p* = 0.0178) and clinical stage (IA/IA/IIb/III/IVA/IVB: 68/7/13/11/0/1 for DFT vs. 66/2/8/24/0/0 5, *p* = 0.023) also showed differences.

**TABLE 1:** Patient demographics and clinicopathological characteristics.

Characteristics	All			Propensity score‐matched			
	DFT (*n* = 100)	UFT (*n* = 105)	*p*‐Value	DFT (*n* = 42)	UFT (*n* = 42)	*p*‐Value	SMD
Age, years (mean ± SE)	68.0 ± 1.1	67.0 ± 1.1	0.50	68.6 ± 1.6	69.5 ± 1.6	0.706	0.083
Sex (male/female, *n*)	86/14	73/32	0.0047	34/8	33/11	0.827	0.096
BMI, kg/m^2^ (mean ± SE)	23.2 ± 0.3	22.9 ± 0.3	0.48	22.5 ± 0.45	22.4 ± 0.5	0.824	0.049
ASA physical status (1/2/3, *n*)	8/83/9	7/87/11	0.89	4/33/5	4/34/4	0.94	0.078
Hb, g/dL (mean ± SE)	13.4 ± 0.2	13.6 ± 0.2	0.48	13.4 ± 0.42	13.2 ± 0.2	0.65	0.10
Alb, g/dl (mean ± SE)	4.2 ± 0.04	4.2 ± 0.04	0.27	4.2 ± 0.06	4.2 ± 0.06	0.83	0.048
Tumor location (PCG/EGJC, *n*)	44/56	52/53	0.42	18/24	20/22	0.66	0.096
Neoadjuvant chemotherapy (yes/no, *n*)	8/92	13/92	0.30	4/38	5/37	0.72	0.077
Surgical approach (laparoscope/robot, *n*)	85/15	32/73	<0.0001	28/14	30/12	0.83	0.10
Type of esophagogastrostomy (end to side/side to side anastomosis, *n*)	22/78	9/96	0.0073	16/26	18/24	0.66	0.097
Clinical T status (cT1/T2/T3/T4a, *n*)	57/18/21/4	60/8/21/16	0.0178	26/7/7/2	27/2/9/4	1.00	0.049
Clinical N status (cN0/cN+, *n*)	81/19	74/31	0.080	36/6	33/9	0.39	0.19
Clinical stage (I/IIA/IIB/III/IVA/IVB, *n*)	68/7/13/11/0/1	66/2/8/24/0/5	0.023	32/1/4/5/0/0	28/1/5/7/0/1	0.79	0.29

Abbreviations: ASA, American Society of Anesthesiologists; BMI, body mass index; DFT, double flap technique; EGJC, esophagogastric junction cancer; PCG, proximal gastric cancer; TNM staging was based on the Japanese Classification of Gastric Carcinoma, 3rd English Edition; UFT, U‐shaped flap technique.

After propensity score matching, each group comprised 42 patients, ensuring comparability across the study arms. No statistically significant differences were observed between the groups in terms of patient characteristics such as age, gender, body mass index, American Society of Anesthesiologists physical status, surgical approaches (laparoscopic/robotic), tumor location, type of esophagogastrostomy, curability, and clinical/pathological Tumor‐Node‐Metastasis stage. The distributions of clinical/pathological T stage, nodal status, and overall tumor stage, as classified by the Japanese Classification of Gastric Cancer (English version, 3rd edition), were also similar.

### 3.2 Surgical outcomes

Table 2 summarizes the intraoperative and postoperative findings for the entire cohort and for the propensity score‐matched groups. In all cohorts, the median operating times and blood loss were comparable between the DFT and UFT groups. Median postoperative hospital stay was shorter for the UFT group (7 days, IQR 7–9) compared to the DFT group (8 days, IQR 7–11), with this difference reaching statistical significance (*p* = 0.014). Early complication rates were comparable between the groups; however, anastomotic leakage was observed in 3% of the double flap technique (DFT) group, while no such instances occurred in the U‐shaped flap technique (UFT) group (*p* = 0.074). Other complications such as wound infection, pancreatic fistula, and pneumonia did not differ significantly in incidence. Late complications such as anastomotic stenosis were similar in both groups. After propensity score matching, significant differences were noted in operating times, with the double flap technique (DFT) group recording longer median times (275 min, IQR 240–333) compared to the U‐shaped flap technique (UFT) group (241 min, IQR 184–315), which was statistically significant (*p* = 0.044). However, there were no significant differences between the two groups in estimated blood loss, number of lymph nodes harvested, incidence of early complications, postoperative hospital stay, and late complications such as anastomotic stenosis. There was no postoperative mortality reported in either group.

**TABLE 2:** Intraoperative and postoperative findings.

Findings	All			Propensity score‐matched		
	DFT (*n* = 100)	UFT (*n* = 105)	*p*‐Value	DFT (*n* = 42)	UFT (*n* = 42)	*p*‐Value
Operating time (min, median [IQR])	292 (242–384)	288 (224–398)	0.82	279 (240–359)	241 (184–345)	0.048
Estimated blood loss (mL, mean ± SE)	30 ± 7	39 ± 7	0.36	24 ± 7	28 ± 7	0.61
Lymph nodes retrieved (*n*, mean ± SE)	35.4 ± 1.8	36.8 ± 1.8	0.58	35.5 ± 2.5	35.1 ± 2.5	0.91
Postoperative hospital stay (days, median [IQR])	8 (7–11)	7 (7–9)	0.014	8 (7–11)	8 (7–9)	0.43
Pathological T status (pT0/T1/T2/T3/T4, *n*)	1/53/17/19/10	0/50/16/32/7	0.43	1/19/8/7/7	0/22/9/10/1	0.25
Pathological N status (pN0/pN+(N1/N2/N3), *n*)	66/34 (15/10/9)	65/39^a^ (18/11/10)	0.48	33/9 (5/2/2/0)	27/14^a^ (7/3/3/1)	0.74
Pathological stage (I/II/III/IV, *n*)	2/64/15/18/1	55/29/13/6^a^	0.05	31/6/5/0	23/12/5/1^a^	0.64
Type of tumor (dif/undif/others, *n*)	72/26/2	71/28/6	0.38	27/15/0	28/12/2	0.31
Curability (R0/R1, *n*)	100/0	99/6	0.015	42/0	40/2	0.15
Early complications^b^ (%)	12 (12%)	11 (10.5%)	0.73	3 (7.1%)	4 (9.5%)	1.00
Grade I						
Wound infection	1	0		0	0	
Grade II						
Pancreatic fistula	1	0		0	0	
Delayed gastric emptying	0	4		0	1	
Intra‐abdominal fluid collection	1	1		0	1	
Pneumonia	2	2		0	1	
Pulmonary embolism	1	0		1	0	
Fever	1	1		0		
Grade III						
Anastomotic leakage	3	0		1	0	
Pleural effusion	1	0		1	0	
Pneumonia	1	0		0	0	
Abdominal incisional hernia	0	1		0	1	
Late complications (%)						
Anastomotic stricture	7 (7%)	7 (6.7%)	0.94	2 (4.8%)	1 (2.4%)	0.57
Postoperative mortality	0	0	1.000	0	0	1.00

*Note*: Anastomotic stricture is indicated by Clavien–Dindo classification grade ≥3, which necessitates endoscopic balloon dilation.

Abbreviations: DFT, double flap technique; Dif: differentiated; Undif: undifferentiated; IQR, Interquartile range; UFT, U‐shaped flap technique.

^a^ Patients with GIST and liposarcoma were excluded.

^b^ Complications graded according to the Clavien–Dindo classification.^22^

### 3.3 Reflux assessment

Table 3 presents reflux symptoms and endoscopic findings 1‐year post‐treatment for both the full cohort and the propensity score‐matched groups. The UFT group reported significantly fewer instances of regurgitation compared to the DFT group (0% vs. 7%; *p* = 0.0058). According to Visick scores, all UFT patients were asymptomatic, whereas the DFT group included more cases graded II/III. Furthermore, the distribution of esophagitis grades, as classified by the Los Angeles (LA) system, significantly differed between the groups (*p* = 0.011). Grade B or higher esophagitis was more prevalent in the DFT group (15%) than in the UFT group (1.9%, *p* = 0.0006). This pattern persisted in the propensity score‐matched analysis, with 14.3% of the DFT group displaying LA grade B or higher esophagitis, compared to none in the UFT group (*p* = 0.0011).

**TABLE 3:** Reflux symptoms and endoscopic findings at 1 year after surgery.

Findings	All			Propensity score‐matched		
	DFT (*n* = 100)	UFT (*n* = 105)	*p*‐Value	DFT (*n* = 42)	UFT (*n* = 42)	*p*‐Value
Reflux symptoms (yes/no)	7/93	0/105	0.0058	2/40	0/42	0.49
Visick score (I/II/III)	93/2/5	105/0/0	0.0022	40/1/1	42/0/0	0.36
Reflux esophagitis, LA grade B or over^a^	15 (15.0%)	2 (1.9%)	0.0006	4 (9.5%)	0 (0%)	0.040
Grade N	71	93		34	41	
Grade M	1	2		1	0	
Grade A	13	8		3	1	
Grade B	8	2		4	0	
Grade C	6	0		0	0	
Grade D	1	0		0	0	

Abbreviations: DFT, double flap technique; UFT, U‐shaped flap technique.

^a^ Reflux esophagitis is classified according to the Los Angeles (LA) classification. The Los Angeles classification grades are Grade N—no endoscopic evidence of esophagitis; Grade M—minimal changes in coloration; Grade A—mucosal damage <5 mm confined to mucosal folds; Grade B—at least one mucosal break >5 mm, not continuous between folds; Grade C—severe esophagitis with continuous mucosal breaks between the tops of two or more mucosal folds that involve <75% of the circumference; Grade D—critical esophagitis with circumferential mucosa damage.

**FIGURE 3:**
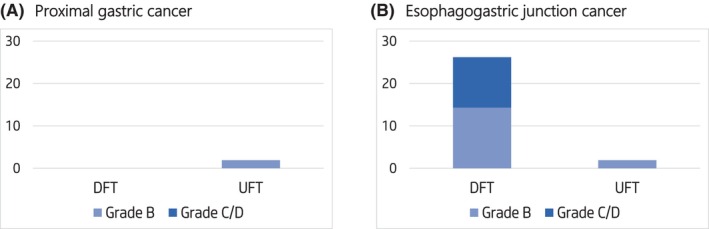
Incidence of Grade B or higher reflux esophagitis according to Los Angeles classification. DFT, double flap technique; UFT, U‐shaped flap technique. The Los Angeles classification grades are Grade B—at least one mucosal break >5 mm, not continuous between folds; Grade C—severe esophagitis with continuous mucosal breaks between the tops of two or more mucosal folds that involve less than 75% of the circumference; Grade D—critical esophagitis with circumferential mucosa damage.

In patients with proximal gastric cancer, there was no significant difference in reflux esophagitis between the DFT and UFT groups (0% vs. 1.9%). However, in patients with esophagogastric junction cancer (EGJC), grade B or higher reflux esophagitis was significantly more frequent in the DFT group (26.8% vs. 1.9% in UFT, *p* = 0.0006). More severe reflux esophagitis (LA‐C or higher) was observed in 14.3% of DFT patients, but not in the UFT group (Figure 3).

### 3.4 Incidence of endoscopic esophageal reflux (Figure 4)

**FIGURE 4:**
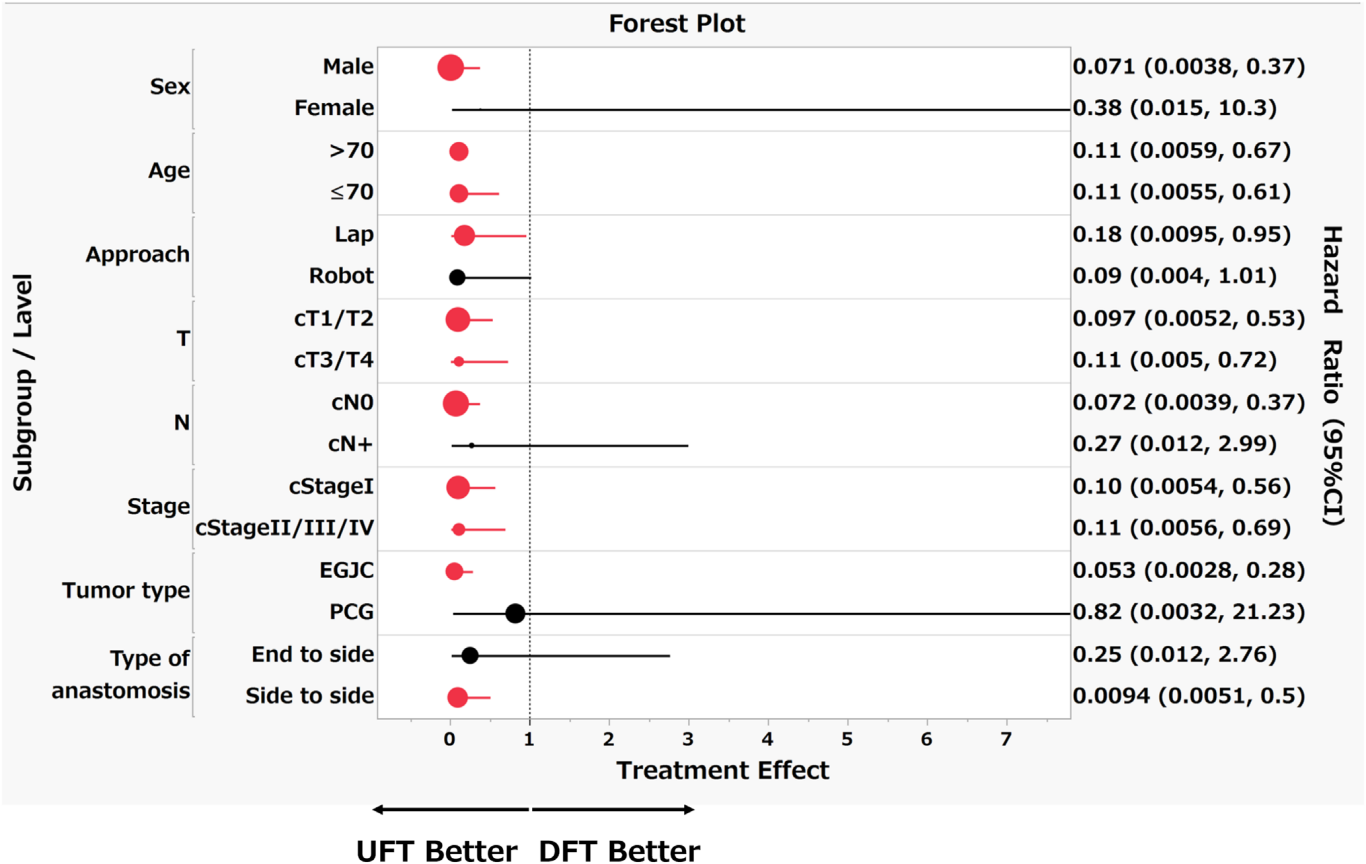
Forest plot of grade B or higher reflux esophagitis in Los Angeles classification.

Figure 4 presents a forest plot analysis comparing the incidence of Los Angeles grade B or higher gastroesophageal reflux disease (GERD) following esophageal reconstruction using the U‐shaped flap technique (UFT) versus the double flap technique (DFT). Forest plot analysis revealed a trend for the U‐shaped flap technique (UFT) to reduce the likelihood of developing postoperative gastroesophageal reflux disease (GERD) in the overall cohort and in subgroups. Particularly notable was the more substantial effect observed in subgroups such as men, patients with esophagogastric junction cancer (EGJC), and those who underwent side‐to‐side anastomosis.

### 3.5 Risk factor analysis

Table 4 summarizes the univariate and multivariate analyses of potential risk factors for developing Los Angeles grade B or higher reflux esophagitis in the entire cohort. The analyses revealed that while age over 70 years did not significantly impact the likelihood of developing reflux esophagitis, male sex and a BMI of 30 kg/m^2^ or higher were associated with an increased risk, although these associations did not reach statistical significance. The type of esophagogastric anastomosis (end‐to‐side vs. side‐to‐side) was associated with a lower risk of reflux esophagitis (OR: 0.21, 95% CI: 0.048–0.67, *p* = 0.0067), although this significance was not maintained in the multivariate analysis (OR: 0.51, 95% CI: 0.11–1.84, *p* = 0.32). Importantly, the type of valvuloplastic esophagogastrostomy (UFT vs. DFT) demonstrated a significant protective effect against reflux esophagitis in both univariate (OR: 0.11, 95% CI: 0.017–0.40, *p* = 0.0038) and multivariate analyses (OR: 0.13, 95% CI: 0.020–0.52, *p* = 0.0024). Additionally, esophagogastric junction (EGJ) cancer was identified as a statistically significant risk factor for the development of Los Angeles grade B or higher reflux esophagitis compared to proximal gastric cancer (PCG), with an OR of 10.04 (95% CI: 2.85–63.67, *p* < 0.0001) in the univariate analysis, and this association remained significant after adjustment for other variables in the multivariate analysis (OR: 12.25, 95% CI: 2.71–263.04, *p* = 0.0005).

**TABLE 4:** Risk factor for Los Angeles grade B or over reflux esophagitis in all cohorts.

	Univariate			Multivariate		
	OR	95% CI	*p* Value	OR	95% CI	*p* Value
Age (>70 years)	1.06	0.48–2.34	0.88			
Sex (male)	2.64	0.88–11.37	0.086			
BMI (≥30 kg/m^2^)	3.94	0.81–15.19	0.084			
Approach (robot)	0.83	0.36–1.82	0.64			
Type of EG (handsewn/stapled)	0.71	0.11–2.67	0.65			
Type of EG (end‐to‐side/side to‐side anastomosis)	0.21	0.048–0.67	0.0067	0.51	0.11–1.84	0.32
Type of VEG (UFT/DFT)	0.11	0.017–0.40	0.0038	0.13	0.020–0.52	0.0024
EGJ cancer/PCG	10.04	2.85–63.67	<0.0001	12.25	2.71–263.04	0.0005

Abbreviations: BMI, body mass index; DFT, double flap technique; EG, esophagogastrostomy; EGJC, esophagogastric junction cancer; PCG, proximal gastric cancer; PNI, prognostic nutritional index; UFT, U‐shaped flap technique; VEG, valvuloplastic esophagogastrostomy.

### 3.6 Nutritional status

There were no significant differences in weight changes observed post‐surgery. Specifically, the results after propensity score matching were as follows: at 3 months post‐surgery, weight change was 89.6% (range 85.0–94.0) for the DFT group and 89.1% (range 86.6–93.1) for the UFT group; at 6 months post‐surgery, 87.6% (range 83.1–94.3) versus 86.8% (range 81.3–93.0); and at 1 year post‐surgery, 87.3% (range 80.3–94.9) versus 86.5% (range 79.8–92.8). Additionally, no significant differences were found in serum albumin, hemoglobin, or prognostic nutritional indicators at 3 months, 6 months, and 1 year post‐surgery.

## 4 DISCUSSION

This study conducted a comparative analysis of the double flap technique (DFT) and the U‐shaped flap technique (UFT) for the surgical treatment of esophageal and gastric junction cancers. Propensity score matching was used to effectively balance baseline characteristics between the two groups, allowing reliable assessment of surgical outcomes and postoperative complications. The results showed that UFT not only resulted in significantly shorter operative time and hospital stay compared to DFT, but also significantly reduced the incidence of postoperative reflux symptoms and high‐grade (LA grade B or over) reflux esophagitis. The UFT's simplified technique reduces the number of operative steps, which can potentially shorten the operative time and reduce the overall complexity of the surgery. The significant reduction in total operation time for the UFT compared to the DFT suggests that the UFT is more efficient, particularly during the reconstruction phase. This is particularly advantageous for enhancing surgical efficiency and minimizing the risk of intraoperative complications. This benefit was particularly pronounced in esophagogastric junction carcinoma (EGJC) patients, with UFT showing fewer instances of reflux esophagitis than DFT. These results indicate a robust anti‐reflux mechanism in UFT, especially valuable in inferior mediastinal reconstruction for EGJC.

Previous studies have shown that inferior mediastinal reconstruction in EGJC is linked to higher complication rates and poorer prognosis, primarily due to the technical complexities involved. A multicenter prospective study reported by Kurokawa et al. highlighted a relatively high anastomotic leak rate (11.9%) in such cases, underscoring the necessity for improved anastomotic techniques.^25^ Kanaji et al. reported a 6% incidence of anastomotic leakage in modified esophagogastrostomy in patients with Siewert type II esophagogastric junction carcinoma (EGJC).^26^ Our study findings suggest comparable or improved operative outcomes, including anastomotic leakage rates, in both the double flap technique (DFT) and U‐shaped flap technique (UFT) groups across all cohorts (3% vs. 0%, respectively, *p* = 0.074). This suggests that the secure wrapping of the anastomotic site and esophagus by the UFT contributes to the reduced complication rate.

Simple esophagogastrostomy (EG) without adjunctive anti‐reflux procedures has been reported to result in reflux esophagitis in 9.1% to 35.3% of patients with proximal gastric cancer (PGC).^27^ Kuroda et al. observed that laparoscopic procedures had higher anastomotic morbidity rates compared to open procedures.^9^ Recent evidence reported by Kuroda et al. in a single‐arm multicenter prospective study evaluated the anti‐reflux efficacy of the laparoscopic double flap technique (DFT) in 40 patients (39 with PCG and one with EGJC).^28^ The study found a stricture rate of 5.3% and an incidence of Grade B or higher reflux esophagitis of 5.3%. This study provides valuable insights into the efficacy of DFT in preventing reflux esophagitis, supporting its use as an effective surgical technique. While the original double flap technique (DFT) offered good surgical outcomes and reliable anti‐reflux function, its implementation within the mediastinal cavity presented significant challenges.^9^ Matsuo et al. identified esophageal invasion exceeding 2 cm as a risk factor for regurgitation and reflux esophagitis in DFT, suggesting that the formation of an adequate pseudofornix may mitigate these risks.^29^ To address these challenges, several stapled anastomosis techniques have been developed, yielding favorable outcomes.^20^, ^21^ Additionally, the precision and reliability of robotic surgery, facilitated by articulated robotic arms, offer promising advances in valvuloplasty esophagogastrostomy.^24^, ^28^, ^30^ To address this, we developed the U‐shaped flap technique (UFT), which may simplify valvuloplastic esophagogastrostomy and effectively shorten the operative time. In our study, multivariate analysis did not identify gender, surgical approach (robotic vs. laparoscopic), or anastomosis technique (hand‐sutured vs. stapled) as risk factors for reflux esophagitis. However, the presence of esophagogastric junction carcinoma (EGJC) and the use of the double flap technique (DFT) were significant risk factors. A multicenter retrospective study conducted by Kuroda et al.^9^ identified anastomosis in the lower mediastinum as a significant risk factor for reflux esophagitis. The study reported an incidence of reflux esophagitis in DFT in the mediastinum of 24.3% for all grades and 18.2% for grade B or higher. Additionally, the incidence of all grades of reflux esophagitis was 37.5% during the early period when the study cohort was divided into two groups based on the period. In our cohort of patients with EGJC, those treated using the DFT exhibited a higher incidence of Grade B or higher reflux esophagitis (26.8%) compared to those treated with the U‐shaped flap technique (UFT) (1.9%, *p* = 0.0006). Additionally, LA Grade C or higher reflux esophagitis occurred in 14.3% of the DFT group but was not observed in the UFT group. These findings suggest that the UFT provides more robust anti‐reflux effects and effectively manages conditions prone to reflux at thoracic anastomoses, especially under negative pressure conditions within the thoracic cavity. Both techniques involve suturing the upper edge of the gastric mucosal window to the esophagus 5 cm oral from the anastomosis, but in the UFT, the flap covers the anastomosis and esophagus over a 5 cm span, resembling a crew‐neck shirt, thereby creating a 5 cm high‐pressure zone. In DFT, the flap covers the esophagus in the form of a low‐cut V‐neck shirt, creating a high‐pressure zone of only 3 cm. This suggests that the UFT may have a better anti‐reflux effect than the DFT because it covers the esophagus 2 cm longer. On the other hand, there was no esophageal transit disorder in the DFT group, which could be caused by over‐tightening the esophagus, and there was no difference in weight loss rate or nutritional status between the two groups.

The U‐shaped flap technique (UFT) aims to enhance anti‐reflux efficacy by embedding the esophagus over a longer distance compared to the double flap technique (DFT). This extended wrapping distance is believed to provide a stronger and more consistent pressure against the esophagus, thereby preventing reflux more effectively. Although the theoretical basis for this approach is strong, currently, there are no specific studies that have demonstrated the benefit of embedding a longer segment of the esophagus for reflux prevention. Our findings suggest that this method is effective, but further research is needed to validate this mechanism with empirical data.

The U‐shaped flap technique introduced in this study demonstrated superior anti‐reflux efficacy compared to the conventional double flap technique (DFT), attributable to both the extended covering area of the lower esophagus and the unique configuration of the flap. The longer covering area provides a larger surface to exert pressure against the esophagus, enhancing the anti‐reflux effect. While extending the covering area in the DFT might also improve its efficacy, but the central incision in the DFT could still compromise the integrity of the anti‐reflux barrier. In contrast, the U‐shaped flap eliminates this central incision, creating a continuous and uniform flap that wraps around the esophagus. This design provides stronger and more consistent pressure, reducing the risk of structural failure or dehiscence and making the U‐shaped flap inherently more robust and reliable. Therefore, the superior performance of the U‐shaped flap in preventing reflux is primarily due to its unique configuration, which offers a secure and durable anti‐reflux barrier, especially in challenging cases such as esophagogastric junction cancer. These findings underscore the importance of both the length of the covering area and the structural integrity of the flap in optimizing anti‐reflux efficacy.

While our study demonstrates the potential benefits of the U‐shaped flap technique (UFT), certain limitations must be addressed. This study was conducted as a single‐institute retrospective study. Although propensity score matching was used to reduce bias, the observational nature of the study cannot completely eliminate it. Additionally, the historical difference between the periods during which the UFT and the double flap technique (DFT) were performed may still affect the outcomes. The DFT was primarily performed earlier, while the UFT was adopted more recently. This temporal difference could introduce a historical bias, as surgical techniques, perioperative care, and other medical advancements may have evolved, potentially influencing the outcomes observed in our study. Future research should concentrate on larger, multi‐center, prospective studies to further validate the benefits of UFT and explore its long‐term outcomes.

In conclusion, our study indicates that the U‐shaped flap technique (UFT) offered significant advantages in reducing postoperative reflux symptoms and endoscopic esophagitis, in a cohort of patients with proximal gastric and esophagogastric junction cancer. The observed lower incidence of anastomotic leakage, reduced operative times, and shorter hospital stays associated with UFT further underscore its potential as the preferred technique in proximal gastrectomy.

## AUTHOR CONTRIBUTIONS

Takeshi Omori: Conceptualization; data curation; formal analysis; investigation; methodology; project administration; writing – original draft; writing – review and editing. Hisashi Hara: Conceptualization; formal analysis; writing – review and editing. Yoshitomo Yanagimoto: Conceptualization; formal analysis; writing – review and editing. Naoki Shinno, Yasunori Masuike, Takashi Kanemura, Hisashi Wada: Data curation; investigation; visualization. Masayushi Yasui, Masayuki Ohue, Hiroshi Miyata: Data curation; investigation; project administration.

## FUNDING INFORMATION

None.

## CONFLICT OF INTEREST STATEMENT

The authors declare no conflicts of interest for this article.

## ETHICS STATEMENT

Approval of the research protocol by an Institutional Reviewer Board: This study design was reviewed and approved by the institutional review board of Osaka International Cancer Institute (Protocol ID: 18070‐3). All procedures performed in studies involving human participants were in accordance with the ethical standards of the institutional and/or national research committee and with the 1964 Helsinki Declaration and its later amendments or comparable ethical standards.

Informed consent: Informed consent, in written form, was secured from all patients. Before undergoing surgery, these patients were thoroughly informed about the associated surgical and oncological risks.

Registry and the Registration No. of the study/trial: N/A.

Animal studies: N/A.

## References

[1] Sung H , Ferlay J , Siegel RL , Laversanne M , Soerjomataram I , Jemal A , et al. Global Cancer Statistics 2020: GLOBOCAN estimates of incidence and mortality worldwide for 36 cancers in 185 countries. CA Cancer J Clin. 2021;71(3):209–249. 33538338 10.3322/caac.21660

[2] Sun KK , Wu YY . Current status of laparoscopic proximal gastrectomy in proximal gastric cancer: technical details and oncologic outcomes. Asian J Surg. 2021;44(1):54–58. 32981822 10.1016/j.asjsur.2020.09.006

[3] Ahn HS , Lee HJ , Yoo MW , Jeong SH , Park DJ , Kim HH , et al. Changes in clinicopathological features and survival after gastrectomy for gastric cancer over a 20‐year period. Br J Surg. 2011;98(2):255–260. 21082693 10.1002/bjs.7310

[4] Xu Y , Tan Y , Wang Y , Xi C , Ye N , Xu X . Proximal versus total gastrectomy for proximal early gastric cancer: a systematic review and meta‐analysis. Medicine (Baltimore). 2019;98(19):e15663. 31083268 10.1097/MD.0000000000015663 PMC6531105

[5] Takiguchi N , Takahashi M , Ikeda M , Inagawa S , Ueda S , Nobuoka T , et al. Long‐term quality‐of‐life comparison of total gastrectomy and proximal gastrectomy by postgastrectomy syndrome assessment scale (PGSAS‐45): a nationwide multi‐institutional study. Gastric Cancer. 2015;18(2):407–416. 24801198 10.1007/s10120-014-0377-8

[6] Nunobe S , Ida S . Current status of proximal gastrectomy for gastric and esophagogastric junctional cancer: a review. Ann Gastroenterol Surg. 2020;4(5):498–504. 33005844 10.1002/ags3.12365 PMC7511558

[7] Hosoda K , Yamashita K , Moriya H , Mieno H , Ema A , Washio M , et al. Laparoscopically assisted proximal gastrectomy with esophagogastrostomy using a novel “open‐door” technique: LAPG with novel reconstruction. J Gastrointest Surg. 2017;21(7):1174–1180. 28025772 10.1007/s11605-016-3341-6

[8] Kuroda S , Nishizaki M , Kikuchi S , Noma K , Tanabe S , Kagawa S , et al. Double‐flap technique as an antireflux procedure in esophagogastrostomy after proximal gastrectomy. J Am Coll Surg. 2016;223(2):e7–e13. 27157920 10.1016/j.jamcollsurg.2016.04.041

[9] Kuroda S , Choda Y , Otsuka S , Ueyama S , Tanaka N , Muraoka A , et al. Multicenter retrospective study to evaluate the efficacy and safety of the double‐flap technique as antireflux esophagogastrostomy after proximal gastrectomy (rD‐FLAP study). Ann Gastroenterol Surg. 2019;3(1):96–103. 30697614 10.1002/ags3.12216 PMC6345660

[10] Hayami M , Hiki N , Nunobe S , Mine S , Ohashi M , Kumagai K , et al. Clinical outcomes and evaluation of laparoscopic proximal gastrectomy with double‐flap technique for early gastric cancer in the upper third of the stomach. Ann Surg Oncol. 2017;24(6):1635–1642. 28130623 10.1245/s10434-017-5782-x

[11] Siewert JR , Hölscher AH , Becker K , Gössner W . Cardia cancer: attempt at a therapeutically relevant classification. Chirurg. 1987;58(1):25–32. 3829805

[12] Omori T , Hara H , Shinno N , Yamamoto M , Kanemura T , Takeoka T , et al. Safety and efficacy of preoperative indocyanine green fluorescence marking in laparoscopic gastrectomy for proximal gastric and esophagogastric junction adenocarcinoma (ICG MAP study). Langenbeck's Arch Surg. 2022;407(8):3387–3396. 36227384 10.1007/s00423-022-02680-9

[13] Omori T , Yamamoto K , Hara H , Shinno N , Yamamoto M , Fujita K , et al. Comparison of robotic gastrectomy and laparoscopic gastrectomy for gastric cancer: a propensity score‐matched analysis. Surg Endosc. 2022;36(8):6223–6234. 35229214 10.1007/s00464-022-09125-w

[14] Japanese Gastric Cancer Association . Japanese classification of gastric carcinoma: 3rd English edition. Gastric Cancer. 2011;14(2):101–112. 21573743 10.1007/s10120-011-0041-5

[15] Japanese Gastric Cancer Association . Japanese gastric cancer treatment guidelines 2021 (6th edition). Gastric Cancer. 2023;26(1):1–25. 36342574 10.1007/s10120-022-01331-8 PMC9813208

[16] Nunobe S , Hayami M , Hiki N . Morphological and functional reconstruction of the esophago‐gastric junction with a double‐flap technique after laparoscopic proximal gastrectomy. Age (Years). 2017;65(2.0):30–89.

[17] Omori T , Moon J‐H , Yanagimoto Y , Sugimura K , Miyata H , Yano M . Pure single‐port laparoscopic proximal gastrectomy using a novel double‐flap technique. Annals of laparoscopic and endoscopic. Surgery. 2017;2: 123.

[18] Saeki Y , Tanabe K , Yamamoto Y , Ohta H , Saito R , Ohdan H . Laparoscopic proximal gastrectomy with hinged double flap method using knotless barbed absorbable sutures: a case series. Int J Surg Case Rep. 2018;51:165–169. 30172056 10.1016/j.ijscr.2018.08.041 PMC6122145

[19] Omori T , Moon JH , Yamamoto K , Yanagimoto Y , Sugimura K , Miyata H , et al. A modified efficient purse‐string stapling technique (mEST) that uses a new metal rod for intracorporeal esophagojejunostomy in laparoscopic total gastrectomy. Transl Gastroenterol Hepatol. 2017;2:61. 28815221 10.21037/tgh.2017.06.01 PMC5539391

[20] Yanagimoto Y , Omori T , Odagiri K , Kawase T , Takeyama H , Suzuki Y , et al. A novel surgical technique for double flap reconstruction using a circular stapler after laparoscopic proximal gastrectomy. J Gastrointest Surg. 2023;27(10):2209–2212. 37674100 10.1007/s11605-023-05822-6

[21] Omori T , Yamamoto K , Yanagimoto Y , Shinno N , Sugimura K , Takahashi H , et al. A novel valvuloplastic esophagogastrostomy technique for laparoscopic transhiatal lower esophagectomy and proximal gastrectomy for Siewert type II esophagogastric junction carcinoma‐the tri double‐flap hybrid method. J Gastrointest Surg. 2021;25(1):16–27. 32157606 10.1007/s11605-020-04547-0

[22] Clavien PA , Barkun J , de Oliveira ML , Vauthey JN , Dindo D , Schulick RD , et al. The Clavien‐Dindo classification of surgical complications: five‐year experience. Ann Surg. 2009;250(2):187–196. 19638912 10.1097/SLA.0b013e3181b13ca2

[23] Onodera T , Goseki N , Kosaki G . Prognostic nutritional index in gastrointestinal surgery of malnourished cancer patients. Nihon Geka Gakkai Zasshi. 1984;85(9):1001–1005. 6438478

[24] Raue W , Ordemann J , Jacobi CA , Menenakos C , Buchholz A , Hartmann J . Nissen versus dor fundoplication for treatment of gastroesophageal reflux disease: a blinded randomized clinical trial. Dig Surg. 2011;28(1):80–86. 21293136 10.1159/000323630

[25] Kurokawa Y , Takeuchi H , Doki Y , Mine S , Terashima M , Yasuda T , et al. Mapping of lymph node metastasis from esophagogastric junction tumors: a prospective Nationwide multicenter study. Ann Surg. 2021;274(1):120–127. 31404008 10.1097/SLA.0000000000003499

[26] Kanaji S , Suzuki S , Yamamoto M , Tanigawa K , Harada H , Urakawa N , et al. Simple and reliable transhiatal reconstruction after laparoscopic proximal gastrectomy with lower esophagectomy for Siewert type II tumors: y‐shaped overlap esophagogastric tube reconstruction. Langenbeck's Arch Surg. 2022;407(5):1881–1890. 35486151 10.1007/s00423-022-02536-2

[27] Nakamura M , Yamaue H . Reconstruction after proximal gastrectomy for gastric cancer in the upper third of the stomach: a review of the literature published from 2000 to 2014. Surg Today. 2016;46(5):517–527. 25987497 10.1007/s00595-015-1185-4

[28] Kuroda S , Ishida M , Choda Y , Muraoka A , Hato S , Kagawa T , et al. A multi‐center, prospective, clinical study to evaluate the anti‐reflux efficacy of laparoscopic double‐flap technique (lD‐FLAP study). Ann Gastroenterol Surg. 2024;8(3):374–382. 38707222 10.1002/ags3.12783 PMC11066497

[29] Matsuo K , Shibasaki S , Suzuki K , Serizawa A , Akimoto S , Nakauchi M , et al. Efficacy of minimally invasive proximal gastrectomy followed by valvuloplastic esophagogastrostomy using the double flap technique in preventing reflux oesophagitis. Surg Endosc. 2023;37(5):3478–3491. 36575220 10.1007/s00464-022-09840-4

[30] Shibasaki S , Suda K , Nakauchi M , Kikuchi K , Kadoya S , Ishida Y , et al. Robotic valvuloplastic esophagogastrostomy using double flap technique following proximal gastrectomy: technical aspects and short‐term outcomes. Surg Endosc. 2017;31(10):4283–4297. 28364148 10.1007/s00464-017-5489-x

